# On the diagnostic and neurobiological origins of bipolar disorder

**DOI:** 10.1038/s41398-020-0796-8

**Published:** 2020-04-23

**Authors:** Alexander W. Charney, Niamh Mullins, You Jeong Park, Jonathan Xu

**Affiliations:** 1 Department of Psychiatry, Icahn School of Medicine at Mount Sinai, One Gustave L. Levy Place, New York, NY 10029 USA; 2 Department of Genetics and Genomic Sciences, Icahn School of Medicine at Mount Sinai, One Gustave L. Levy Place, New York, NY 10029 USA; 3 Department of Neuroscience, Icahn School of Medicine at Mount Sinai, One Gustave L. Levy Place, New York, NY 10029 USA; 4 Department of Neurosurgery, Icahn School of Medicine at Mount Sinai, One Gustave L. Levy Place, New York, NY 10029 USA; 5 Mental Illness Research, Education, and Clinical Center (VISN 2 South), James J. Peters Veterans Affairs Medical Center, Bronx, NY 10468 USA

**Keywords:** Molecular neuroscience, Personalized medicine

## Abstract

Psychiatry is constructed around a taxonomy of several hundred diagnoses differentiated by nuances in the timing, co-occurrence, and severity of symptoms. Bipolar disorder (BD) is notable among these diagnoses for manic, depressive, and psychotic symptoms all being core features. Here, we trace current understanding of the neurobiological origins of BD and related diagnoses. To provide context, we begin by exploring the historical origins of psychiatric taxonomy. We then illustrate how key discoveries in pharmacology and neuroscience gave rise to a generation of neurobiological hypotheses about the origins of these disorders that facilitated therapeutic innovation but failed to explain disease pathogenesis. Lastly, we examine the extent to which genetics has succeeded in filling this void and contributing to the construction of an objective classification of psychiatric disturbance.

## Introduction

Psychiatry is currently constructed around a taxonomy of ~300 discrete diagnoses codified in the fifth edition of the Diagnostic and Statistical Manual (DSM-5)^1^ produced by the American Psychiatric Association (APA) and the 10th version of the International Classification of Disease (ICD-10)^2^ produced by the World Health Organization^1^. Diagnoses are delineated by specific criteria for the timing, co-occurrence and severity of symptoms. The DSM-5 defines psychotic, manic, depressive, cognitive, and communicative symptom classes (Table 1). A given symptom may be part of multiple classes. For example, the inability to experience pleasure is both a depressive and a psychotic symptom in the DSM-5. As a result, two patients with the same set of symptoms may receive different diagnoses and the primary diagnosis carried by a patient often changes over time^3^. With each revision of the taxonomy, some diagnoses are removed, new diagnoses are defined, and diagnostic criteria are modified^1,4,5^. Driving the taxonomic enterprise is the sense that discrete mental illnesses exist in nature independent of human knowledge^6^.

**Table 1:** Symptom classes according to diagnostic criteria.

Diagnosis	Symptom classes according to diagnostic criteria				
	Psychotic	Manic	Depressive	Cognitive	Communication
Bipolar I disorder	Possible	Required	Possible	Possible	Possible
Bipolar II disorder	Possible	Required	Required	Possible	Possible
Schizoaffective disorder, bipolar	Required	Required	Possible	Possible	Possible
Schizoaffective disorder, depressive	Required	Possible	Required	Possible	Possible
Schizophrenia	Required	Possible	Possible	Possible	Possible
Major depressive disorder	Possible	Possible	Required	Possible	Possible
Autism spectrum disorder	Possible	Possible	Possible	Possible	Required
Intellectual disability	Possible	Possible	Possible	Required	Possible
Major neurocognitive disorder	Possible	Possible	Possible	Required	Possible

Status with respect to five primary symptom classes for nine DSM-5 diagnoses according to the formal diagnostic criteria. The status describes whether symptoms in the given class must be present for the diagnosis to be made (“Required”) or may be present but not necessary for the diagnosis to be made (“Possible”).

Bipolar disorder (BD) is notable amongst psychiatric diagnoses for manic, depressive, and psychotic symptoms all being core features. Canonically, BD is described as alternating episodes of mania and depression. In reality, individuals who receive this diagnosis are clinically heterogeneous, varying with respect to symptomatology^7,8^, comorbidity^9^, and longitudinal course^10^. There are several diagnoses on the BD spectrum^1,2^, including bipolar I disorder (BD I), bipolar II disorder (BD II), and schizoaffective disorder bipolar type (SAB). The criteria for these diagnoses differ from one another—and from clinically related diagnoses such as schizophrenia (SCZ) and major depressive disorder (MDD)—by nuances in the timing, co-occurrence, and severity of manic, depressive, and psychotic symptoms. The DSM establishes thresholds to differentiate full-blown “episodes” (e.g., manic episodes, depressive episodes) from the sub-threshold occurrence of symptoms that comprise such episodes—for example, “three (or more) of the following symptoms”^1,2^. An episode of mania equates to a diagnosis of BD I unless psychotic symptoms occur both during the episode and for at least 2 weeks outside the episode—if these two criteria are met, then the diagnosis is SAB. A history of hypomanic and depressive episodes equates to a diagnosis of BD II. However, if psychosis occurs during an otherwise hypomanic episode, then the episode is defined as manic and the diagnosis BD I (or SAB if the aforementioned criteria are met). Psychosis during a depressive episode does not preclude a diagnosis of BD II. Manic symptoms do not preclude diagnoses of MDD or SCZ if below threshold for a manic or hypomanic episode. As with other DSM diagnoses, the criteria for disorders on the BD spectrum can be fulfilled in many thousands of different ways^11,12^.

Here, we present a perspective on the current state of knowledge about the pathogenesis of BD and related diagnoses. We have organized this work into three sections. First, we trace the history of psychiatric taxonomy to learn how these diagnoses came to be defined. Second, we review how key advances in neuroscience and pharmacology forged a generation of influential but ultimately inadequate hypotheses regarding their neurobiological origins. Third, we examine the extent to which genetics has succeeded in filling this void and contributing to the construction of an objective classification of psychiatric disturbance.

### Tracing the origins of current psychiatric diagnoses

Psychiatric taxonomy can be traced back to the start of the written record. The earliest known medical texts date to the second millennia before the common era (BCE) in Egypt and Babylonia (present-day Iraq)^13,14^. These texts contain descriptions of psychiatric symptoms but do not define discrete diagnostic entities. Many of the symptoms bear similarity to those in the current taxonomy, such as delusions, hallucinations, compulsions, panic, and depressed mood^13–15^. In the first millennia BCE the oldest known medical texts from China, India, and Greece were written. In these, health is conceptualized as resulting from a harmonious balance of internal forces. In the Chinese texts these forces are abstract ideas (i.e., the yin and the yang)^16^, while in the Indian and Greek texts they are bodily fluids (i.e., humors)^17^. In contrast to the Egyptian and Babylonian texts, in the first millennia BCE symptoms are grouped into discrete diagnostic entities. For instance, Dube reports in the ancient Indian texts 24 mental illnesses formally delineated, including conditions resembling the depressive, psychotic, cognitive, personality, and substance use disorders of the current taxonomy^18^. From the Greek texts of this era the current taxonomy evolved—specifically, from two concepts these texts introduced: *mania* and *melancholia*^2^.

From the time of their inception until over a thousand years later, the terms *mania* and *melancholia* were used broadly to refer to overactive and underactive mental states, respectively^19^. The 2nd century writings of Arataeus illustrate their usage. In some passages, *melancholic* patients are described in terms reminiscent of the DSM-5 diagnostic criteria for MDD: “avoidance of the haunts of men, vain lamentations; they complain of life, and desire to die.” In other passages, though, they are described in terms reminiscent of the DSM-5 definition of the negative symptoms of SCZ: “insensibility and fatuousness, … they become ignorant of all things, or forgetful of themselves, and live the life of the inferior animals”^20^. Ancient usage of the term *mania* was even more heterogeneous. Arataeus described it as having “infinite” variations, united only in that all constitute “chronic derangements of the mind, without fever”^20^.

At the beginning of the Renaissance in the 14th century, psychiatric taxonomies in Europe expanded beyond *mania* and *melancholia*. The term *insanity*, for instance, was introduced by Paracelsus, who differentiated it from *mania* by the absence of paroxysms (i.e., sudden worsening of symptoms)^21^. Prominent voices in medicine in the first half of the 17th century advocated for a comprehensive classification of human disease^22^. In the mid-18th century de Sauvages put forth such a classification in which he categorized over 2400 conditions, cementing the notion of a precise taxonomy as being fundamental to the practice of medicine^23,24^. A great number of psychiatric taxonomies followed. Works from this time introduced many of the diagnostic concepts in use today, with contributions from Cullen^25^, Pinel^26^, Battie^27^, Esquirol^28^, Georget^29^, Griesinger^30^, Bayle^31^, Falret^32^, Baillarger^33^, Morel^34^, Kahlbaum^35^, and many more. These taxonomies were based on the author’s clinical experience and built around the element of mental illness he considered most important (e.g., etiology, anatomy, symptomatology, and disease course). Disagreements arose. Falret and Baillarger, for instance, feuded publicly over who was first to describe the condition today known as BD^36^. Diagnostic clarity remained elusive. Pinel, writing 50 years after de Sauvages, described four classes of mental illness yet acknowledged they were often “mutually interchangeable”^26^. Little had changed 50 years after Pinel, with one participant in a seminal 1860 debate on psychiatric taxonomy lamenting of “patients floating between two classes”^37^. The clinical overlap across diagnoses in the current taxonomy echoes these earlier observations (Table 1).

Amidst the surge in psychiatric taxonomies Kraepelin in the late 19th century began work that has come to be considered the forerunner of the current taxonomy^38^. He systematically characterized the initial presentation and disease course of a hospitalized psychiatric patient cohort^39^. Data were collected on specially designed index cards over 4 weeks, and patients were followed longitudinally after discharge. Kraepelin observed that patients with a variety of initial presentations (such as *mania* and *melancholia*) ultimately progressed to *dementia* (in his words, “the destruction of the personality”)^39^. He concluded a single-disease process was occurring in these patients and sought variables in the initial presentation to predict the outcome^39^, eventually dichotomizing these patients into those who come to exhibit “mental deterioration” (i.e., dementia praecox) and those who do not (i.e., manic-depressive insanity)^40^. Through the application of such approaches over his career a taxonomy took shape that was putatively more objective than those of his predecessors and contemporaries. His final essays, however, betray skepticism toward his primary conclusions. For example, with respect to the dichotomy of dementia praecox and manic-depressive insanity he wrote: “It is becoming increasingly clear that we cannot distinguish satisfactorily between these two illnesses and this brings home the suspicion that our formulation of the problem may be incorrect”^41^.

In the early 20th century, the organization of psychiatrists in the United States (USA) that would later become the APA was asked to develop a taxonomy for use in the federal census. In 1918, they issued the Statistical Manual for the Use of Institutions of the Insane (SM). In this document 22 categories of mental illness were defined. The focus was on severe cases of mental illness where the cause of the disturbance was known (e.g., brain tumors, syphilis, and head trauma)^42^, as at the time psychiatry was primarily concerned with treating such cases. After the Second World War, under the influence of psychoanalytic principles being embraced by society, the primary focus of psychiatry turned to improving mental health in the general population^43^. The shift prompted the APA to reformulate the SM into a more extensive taxonomy centered around psychoanalytic concepts, which they published in 1952 as the DSM (DSM-I)^44^.

In parallel, some US psychiatrists carried forward the work of defining diagnostic criteria through empiric investigation. These efforts culminated with the 1972 publication of a set of 20 diagnoses now referred to as the Feighner criteria^45^. Included were diagnoses of depression, mania, and SCZ that are the direct predecessors to MDD, BD, and SCZ in the current taxonomy. The Feighner criteria for depression and mania evolved from Kraepelin’s definition of manic-depressive insanity by way of Leonhard, who in his 1957 taxonomy split manic-depressive insanity into unipolar (i.e., episodes of depression or mania) and bipolar (i.e., alternating episodes of depression and mania) types^46^. The Feighner criteria for SCZ was a direct descendant of Kraepelin’s definition of dementia praecox (SCZ was the term the psychoanalyst Bleuler used for his modification of dementia praecox^47,48^). In their concluding remarks, Feighner et al. expressed how they would like their work to be viewed in the annals of psychiatric taxonomy: “What we now present is our synthesis of existing information, a synthesis based on data rather than opinion or tradition”^45^. This statement signaled a sentiment growing in US society by then that psychoanalysis—and, by extension, psychiatry in general—was a nonmedical, nonscientific enterprise^49^. In response, the APA made a strategic decision with the third edition of the DSM (DSM-III) to move toward a more objective taxonomy modeled after the Feighner criteria. Notably, DSM-III defined several hundred diagnostic entities beyond those in Feighner et al.

Diagnostic criteria are in a perpetual state of revision. As noted above, Kraepelin’s dichotomy was based on the presence or absence of “mental deterioration” over time^40^ yet in the current taxonomy clinical course is not what differentiates SCZ from BD^1^. Similarly, the original description of schizoaffective disorder by Kasanin featured individuals most notable for being high functioning compared with other psychotic patients^50^, but this is not a criterion for the diagnosis today^51^. The forces shaping these changes are difficult to trace, but scientific knowledge has played a negligible role compared with the tremendous influence of culture, history, and expert opinion^49,52^. As a case in point, consider how five was determined to be the number of symptoms required to receive a diagnosis of MDD in the DSM-5^1^. The threshold is a holdover from the Feighner criteria for depression, where five was selected based on a paper published 15 years earlier by Cassidy et al (who used a threshold of six)^53^. Asked how he determined the threshold, Cassidy explained: “It sounded about right”^54^.

### Early neurobiological hypotheses of BD and related diagnoses

For as long as there have been efforts to classify mental illnesses, there have been efforts to understand their biological foundations. Hippocrates, writing in the 4th and 5th centuries BCE, recognized the brain as the seat of mental function: “From nothing else but the brain come joys, delights, laughter and sports, and sorrows, griefs, despondency, and lamentations”^55^. Modern conceptions of the biology of mental illness began to take shape by the start of the Renaissance. In the early 16th century, for instance, Paracelsus described the complex patterns of inheritance characteristic of mental illness. He observed it may be “received … as a heritage” from parents who “may or may not be insane” and “it may even happen that if both are insane they still would give birth to a healthy child”^21^. Weyer, a contemporary of Paracelsus, formulated an early neurobiological explanation of depressed mood. Of patients with *melancholia*, he stated: “You will recognize to what extent all their senses are deprived by the melancholic humor which is spread over their brains”^56^.

In the 19th century, the central nervous came to be better understood. Seminal experiments toward this end by Vulpian concluded that the adrenal medulla synthesized an unknown substance circulating throughout the body^57^ that Oliver and Schäfer later discovered caused a rise in arterial blood pressure^58^. The therapeutic implications of this finding stimulated a search for the active ingredient, and in 1901 Takamine patented “adrenalin”^59^. Over the next 30 years, the work of many scientists led to the synthesis and purification of compounds with similar structural and/or physiological properties, including dopamine^60^ and serotonin^61^. By the mid-20th century, it had been determined such compounds (neurotransmitters) exert clinical effects via cells of the nervous system, attaching to receptors on target cells and orchestrating the flow of electricity across cell membranes^62^. With this progress in neuroscience, a new era in psychopharmacology arose. In the 1940s, Cade identified incidentally that lithium made guinea pigs lethargic, prompting him to administer it to 19 psychiatric patients (10 manic patients, 6 patients with dementia praecox, and 3 melancholics). He found lithium treated the “excitement” symptoms of mania, with no effect on hallucinations, delusions or depression^63^. Around the same period, chlorpromazine was reported as the first effective antipsychotic^64^ and monoamine oxidase inhibitors as the first effective antidepressants^65^. These pharmacological observations were (and continue to be) viewed as evidence that each diagnosis in the psychiatric taxonomy has a unique neurobiological basis. For example, in concluding his initial report on lithium, Cade noted: “The effect on patients with psychotic excitement—that is, true manic attacks—is so specific that it inevitably leads to speculation of a deficiency in the body of lithium ions in the genesis of this disorder”^63^.

With the advent of the radio-ligand binding assay in the 1960s, it became possible to quantitatively evaluate the impact of medications on the activity of neurotransmitters^66^. The basics of this assay are as follows: a brain specimen that contains a receptor of interest is incubated with a known radio-labeled ligand under different conditions (e.g., with and without drug), and the ligand binding activity is then compared^67^. When paired with clinical observations in response to drug administration, the results of such experiments gave rise to a host of single-neurotransmitter/single-disease hypotheses that continue to dominate thinking on the neurobiology of mental illness. In fact, beginning with the catecholamine hypothesis of BD (CHBD)^68^ and the dopamine hypothesis of SCZ^69^, causality has since been proposed for most neurotransmitter/diagnosis combinations: abnormalities in dopaminergic, adrenergic, noradrenergic, serotonergic, glutamatergic, GABA-ergic, and cholinergic neurotransmission have all been posited as the underlying cause of depressive, anxiety, psychotic, manic, and neurodevelopmental symptoms^68–90^.

The lack of empiric support characteristic of these hypotheses is exemplified by the CHBD, which we have evaluated exhaustively elsewhere^91^ and here highlight only points salient for the present discussion. The central principle of the CHBD is that the two clinical poles of BD—mania and depression—result from functional changes in the activities of catecholamines, with low activity causing the depressed state and high activity the manic state^68^. The CHBD emerged in the mid-1960s amidst pharmacological observations that drugs with catecholamine-enhancing properties elevated mood while catecholamine-depleting agents caused depression^68,92,93^. Over time, new iterations of the CHBD have strayed from the original, primarily with regard to which catecholamine is associated with which mood state^94–97^. Many pharmacological probes have been used to indirectly test the CHBD, including L-dopa, dopamine agonists, amphetamines, yohimbine, antidepressants, mood-stabilizers, tyrosine depletion, and AMPT. Considered as a whole^91^, this body of literature underscores the important distinction between drug mechanisms and disease pathogenesis. That is, there is strong evidence that the clinical symptoms of BD can be mimicked and alleviated by pharmacologic modulation of the catecholamine system, but there is no direct evidence that the catecholamine system is involved in the pathogenesis of BD. The same conclusion has been drawn from assessments of other influential hypotheses^98,99^. Pharmacological observation may lead to therapeutic innovation but is insufficient for mapping the neurobiological foundations of psychiatric disturbance.

### Genetic architecture of BD and related diagnoses

In this final section, we cover three principles resulting from psychiatric genetic research. First and foremost, BD and related diagnoses have a genetic basis. Second, they are not caused by a single abnormal gene but rather have a highly polygenic architecture that is not specific to a particular diagnosis. Third, genetic variation partly explains the clinical heterogeneity within—and the clinical overlap between—diagnoses.

Large pedigree studies show that BD aggregates in families. The relative risk for first-degree relatives of BD patients is ~7–10^100,101^. The concordance rate for monozygotic twins is higher (0.5–0.6) than for dizygotic twins (0.39–0.43), giving heritability estimates (79–93%)^102–104^ that suggest genetic variation explains a large component of risk. BD I seems to aggregate in families more so than BD II^105,106^, and familial aggregation of several sub-phenotypes within BD has been observed (e.g., early age-of-onset, psychosis)^100^. BD co-aggregates in families with related diagnoses such as MDD^107^ and SCZ^108,109^, with SCZ more frequently co-aggregating with BD I compared with BD II^110^ and MDD not clearly co-aggregating with a particular BD subtype^111,112^. Relatives of SAB probands appear to have relatively equivalent risk for SAB, SCZ, and BD^113,114^. These observations led to searches for responsible genes.

Linkage and candidate gene studies dominated psychiatric genetics research for several decades beginning in the 1980s. The central idea of linkage analysis is that high co-occurrence of a genotype and a trait suggests the genotype is “linked” to a chromosomal segment harboring a variant that causes the trait. To perform this analysis hundreds of markers across the genome must be genotyped in families where a trait of interest aggregates. This strategy is effective for traits caused by a single genomic locus but led to inconsistent results for BD and related diagnoses, suggesting no single locus accounts for a substantial portion of genetic liability for these conditions^115–117^. Candidate gene studies of BD and related diagnoses have been performed primarily for genes featured in neurobiological hypotheses derived from pharmacological observations, such as *BDNF, COMT, 5HTT*, and *MAOA*. Single nucleotide polymorphisms (SNPs) in the candidate gene are tested for differences in allele frequencies between case and control groups. As with linkage studies, results from these experiments were inconsistent for BD and related diagnoses^118–122^.

With the advent of high-resolution SNP microarrays, it became possible to genotype hundreds of thousands of SNPs at low cost and carry out population-scale genome-wide association studies (GWAS) of BD and related diagnoses. GWAS is often characterized as “hypothesis-free” because SNPs across the entire genome are tested for association with the trait of interest without prior assumptions about which genes or genetic variants are likely to be involved. SNPs included are selected for being common in the population (e.g., minor allele frequency >1%) and include not only SNPs genotyped directly on the microarray but millions of additional SNPs where the genotype can be imputed based on linkage disequilibrium (LD) with the SNPs on the microarray. To reduce the risk of chance positive results when conducting many statistical tests, the conventional genome-wide significance threshold (*p* value < 5 × 10^−^^8^) accounts for the estimated number of independent common SNPs in the genome^123^. SNPs significantly associated with a trait of interest may be functionally relevant or may represent loci which are transmitted in LD with a causative polymorphism. The statistical power of a GWAS is a function of the frequency and effect sizes of the alleles tested, sample size, and the specified significance threshold. There are >10 published BD GWAS. The earliest did not identify SNPs reaching genome-wide significance, likely due to limited sample sizes^124^. As sample sizes increased, genome-wide significant associations with small effect sizes were identified in several genes, including *ANK3* and *CACNA1C*^125–129^. The largest BD GWAS to date included ~30,000 cases and 170,000 controls and found SNPs in 30 independent genomic loci surpassing genome-wide significance. Similar observations have emerged from GWAS of related diagnoses such as SCZ^130^ and MDD^131^. Efforts are underway to better understand the biological mechanism underlying these statistical observations^132–134^. Collapsing diagnoses into a single case cohort (e.g., BD/SCZ, BD/MDD) for comparison to controls consistently yields genetic associations beyond those identified in GWAS of single diagnoses^135–137^—illustrating a shared, cross-diagnostic genetic architecture.

Genome-wide SNP data have been interrogated to determine the extent to which genetics accounts for the clinical heterogeneity within—and similarity between—diagnoses. Several groups have approached this question through SNP-based heritability (SNP-h^2^) and genetic correlation (*r*_*g*_) analyses. Conceptually, SNP-h^2^ represents the proportion of variance in disease status explained by the additive effect of common SNPs, while r_g_ represents the fraction of SNP-h^2^ that is shared between two traits. The most recent estimates of SNP-h^2^ for BD are ~30%^138^. The discrepancy between this estimate and the heritability estimated from pedigree studies is consistent with observations across human traits, where SNP-h^2^ generally accounts for one- to two-thirds of the heritability estimated from pedigrees^139^. Several factors may account for this “missing heritability,” including over-estimation of heritability from pedigrees, insufficient sample size for accurate estimation of SNP-h^2^, and incomplete tagging of rare causal variants by common SNPs^139^. SNP-h^2^ estimates are significantly higher in BD I compared with BD II, mirroring the epidemiological findings that BD I aggregates in families more so than BD II. BD I and BD II have a *r*_*g*_ of ~0.80, significantly lower than the perfect correlation observed between BD cohorts matched for subtype composition^129^. High *r*_*g*_ is observed between BD and both SCZ and MDD^140,141^. Polygenic risk scores (PRS) summarize an individual’s genetic liability for a given trait and have also been used to dissect the shared genetic etiology between BD and related diagnoses. PRS are typically calculated as the weighted sum of genome-wide risk alleles for the trait of interest, where weights are the effect size found for the allele in an independent GWAS of the trait. PRS for SCZ and MDD are higher in BD cases compared with controls^138,142,143^, suggesting risk variants are generally not diagnosis specific. Importantly, the shared architecture between diagnoses is a function of the class of genetic variant under study. For illustration, consider the diagnoses BD, SCZ, and autistic spectrum disorder (AUT). The *r*_*g*_ between BD and SCZ calculated from common SNPs (0.79) is higher than that for BD/AUT (0.04) and SCZ/AUT (0.14)^141,144^. In contrast, studies of rare CNVs have found robust overlapping associations between SCZ and AUT^145^, but not BD^146^.

The shared genetic architecture among diagnoses with shared clinical features has led researchers to assess whether genetic risk occurs at the level of symptoms rather than diagnosis. Amongst BD subtypes, the loading of common SCZ risk variants follows the prominence of psychotic symptoms: SAB cases have the highest SCZ PRS followed by BD I then BD II^129,138,147^, and BD cases with psychosis have higher SCZ PRS than those without^135,146,148^. Likewise, the loading of common MDD risk variants follows the prominence of depressive symptoms, with higher MDD PRS in BD II compared with BD I and SAB^129,137,138^. Efforts to integrate common and rare variant data with high-dimensional clinical data suggest that different classes of genetic variation may contribute to different symptom profiles. For example, our group found that SAB cases have significantly higher CNV burden and SCZ PRS compared with BD I cases regardless of whether BD I cases have a history of psychosis, whereas BD I cases with psychosis compared with those without have a higher SCZ PRS but no CNV burden^146^. One interpretation of these observations is that common SCZ risk alleles contribute to psychosis whereas rare SCZ risk alleles contribute to other domains of psychopathology.

## Perspective

**Fig. 1:**
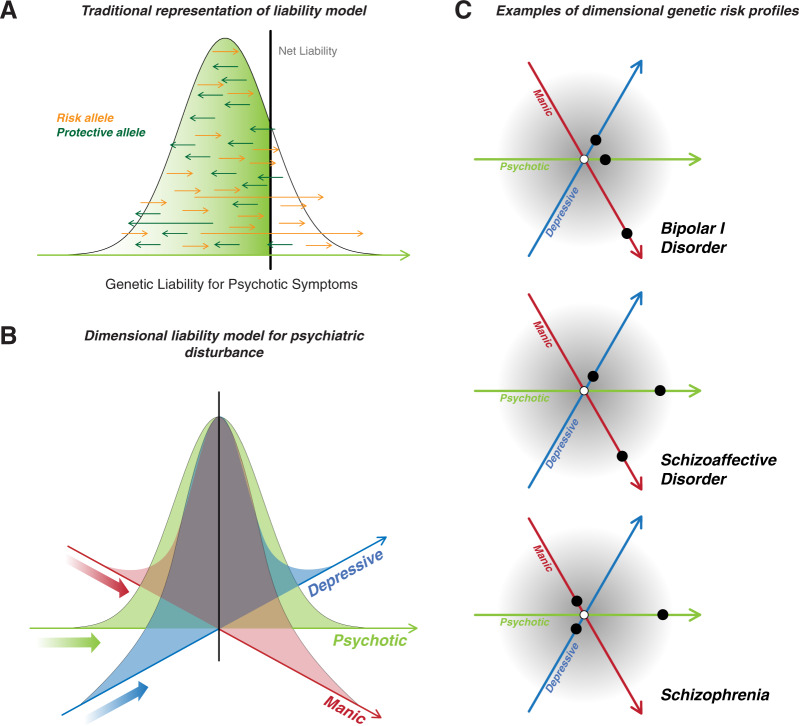
Dimensional liability to psychiatric disturbance. **a** The liability model for complex genetic traits posits that all individuals fall along a spectrum of genetic risk (*x*-axis), where different classes of genetic variation contribute to susceptibility for a given trait (in this example, psychotic symptoms). Under this model, the clinical trait is observed only when the net liability passes a threshold (represented here by the thick vertical black line). **b** The dimensional liability model builds on the traditional liability model to incorporate the observation that genetic risk for symptoms dimensions spans diagnostic categories. Three symptoms dimensions are used in this illustration: depressive (blue), psychotic (green), and manic (red). **c** Under the dimensional liability model, an individual has a genetic liability for each symptom dimension, and the combination of liabilities influences the clinical presentation. We illustrate this concept with three hypothetical individuals: individual 1 (top), individual 2 (middle), and individual 3 (bottom). Colors correspond to the same symptom dimensions as in **b**. The genetic liability for a given symptom dimension is represented as a colored black circle. Individual 1 has high genetic liability in the manic dimension only. Individual 2 has high genetic liability in the depressive and psychotic dimensions. Individual 3 has high genetic liability in the psychotic dimension only. The corresponding clinical presentations in these individuals would, under the dimensional liability model, reflect the combination of genetic loadings for the different dimensions. In this case, individual 1 would present in a manner consistent with a diagnosis of BD I, individual 2 in a manner consistent with schizoaffective disorder bipolar type (or BD I with psychosis), and individual 3 with schizophrenia.

The historical record shows the taxonomy that forms the basis of clinical practice and research in psychiatry lacks an empirical foundation. Most hypotheses about the neurobiological origins of the diagnoses defined in this taxonomy have focused on particular neurotransmitter systems. Tests of these hypotheses have shown that modulating neurotransmitter activity can modulate psychiatric symptoms but cannot provide insight into the pathogenesis of mental illness^91,98^. Genetics has proven valuable as a means by which to assess the validity of diagnostic boundaries and gain direct insight into neurobiological origins. The genetic architecture of mental illness does not adhere to the boundaries delineated in the current taxonomy. One reading of the current literature is that different classes of genetic variation form a diathesis for different classes of psychiatric symptoms, and an individual’s unique combination of risk variants and environment results in his or her unique clinical presentation (Fig. 1). Encouragingly, there is evidence of a partial overlap between the genetic foundations of psychiatric disease and the mechanisms by which existing treatments work^149,150^. It must be acknowledged, however, that the immense progress in psychiatric genetics in the last 10 years has not led to an influx of experimental therapeutics^151^. This is likely due in part to the unfortunate reality that the sheer complexity of the genetic architecture has rendered it difficult to determine which genes (if any) are the central drivers of pathogenesis and thus promising drug targets. Confronting this singular challenge must be the top priority for the field of psychiatric genetics moving forward. These efforts must include not only multiomic data integration to fully dissect the biological mechanisms by which genetic variants increase risk (i.e., through their influence on the transcriptome and epigenome), but also bold, immediate development of experimental therapeutics based on the existing albeit incomplete knowledge of genetic architecture.

To objectively delineate the natural kinds of mental illness will require imaginative applications of the technology of our time. In the 21st century, we still grapple with the basic question of whether the differences between two psychiatric patients outweigh their similarities enough to warrant assigning them different diagnoses. Kraepelin foresaw this stalemate in his final essays^152^, stating: “It is unreasonable to assume that simple clinical observation of patients will eventually lead to far-reaching discoveries.” The genetic overlap across diagnoses is direct, objective evidence that our current taxonomy does not capture truly discrete disease entities. This insight was achieved through large consortia in psychiatric genetics collaborating deeply on an unprecedented global scale. To determine if discrete mental illnesses exist in nature, we must perform deeper characterization of patients at the same scale, integrating genomics with high-resolution, longitudinal clinical data, and other tools for human brain research. Kraepelin also foresaw this as the road to success^152^, hypothesizing that the “delineation of natural disease groups” could be achieved by integrating “all those auxiliary sciences whose aim is to penetrate the core of mental disease processes” and listing as examples sciences concerned with the “laws of heredity” and “the anatomical basis of individual disease processes.” He concluded: “It is natural to turn away from arranging illnesses in orderly well-defined groups and to set ourselves instead the undoubtedly higher and more satisfying goal of understanding their essential structure.”

## Footnotes

1 For all intents and purposes, the DSM-5 and ICD-10 systems are the same with respect to mental illness. For the sake of clarity, therefore, here we trace the origins of only one of them—the DSM-5.

2 Several terms used historically in psychiatric classifications are in use today but have entirely different meanings. In order to distinguish these from one another, we will use italics whenever referring to a term with respect to its historic usage.
